# Laparoscopic nephron-sparing surgery for complex renal cystic lesions: a single-center experience

**DOI:** 10.3389/fonc.2024.1398347

**Published:** 2024-05-28

**Authors:** Dexin Dong, Yushi Zhang

**Affiliations:** Department of Urology, Chinese Academy of Medical Sciences, Peking Union Medical College Hospital, Beijing, China

**Keywords:** complex renal cystic lesions, laparoscopic nephron sparing surgery (LNSS), laparoscopic ultrasonography (LUS), classification, Bosniak

## Abstract

**Objectives:**

This study aimed to explore the feasibility and safety of laparoscopic nephron-sparing surgery (LNSS) for complex renal cystic lesions.

**Methods:**

A retrospective study was conducted on 83 cases of complex renal cystic lesions treated with LNSS in our hospital. There were 32 men and 51 women, ranging in age from 24 to 73 years (average, 47.22 ± 9.03 years). The diameter of the cysts was 1.5–5.9 cm (average, 3.44 ± 0.86cm). According to the Bosniak classification, there were 15 cases of type II, 23 cases of type IIF, 29 cases of type III, and 16 cases of type IV complex renal cystic lesions. According to clinical classification based on the difficulty of laparoscopic partial nephrectomy and the depth of the lesion, the 83 complex renal cystic lesions were divided into 48 cases of the extra-renal type, 15 cases of the centrally located type, seven cases of the renal sinus type, and 13 cases of the renal hilum type.

**Results:**

Laparoscopic partial nephrectomy was successful in all 83 patients. The surgical time was 35–102 min (average, 52.13 ± 14.38 min), the intraoperative bleeding volume was 10–200 ml (average, 27.25 ± 12.26 ml), and the renal artery occlusion time was 12–28 min (average, 12.46 ± 4.45 min). There was no significant change in creatinine before and after surgery. The postoperative pathological results showed 71 cases of renal clear cell carcinoma, five cases of low malignant potential multilocular cystic renal tumors, and seven cases of pure renal cysts with all margins negative.

**Conclusions:**

There is potential for the malignant transformation of complex renal cysts into renal cell carcinoma. For complex renal cysts classified as Bosniak IIF or higher, surgical intervention is recommended, and LNSS is safe and effective. The complexity of the surgical procedure varies depending on the location classification of the complex renal cysts.

## Introduction

With the development of laparoscopic techniques, laparoscopic nephron-sparing surgery (LNSS) has gradually become a standard procedure for early-stage renal tumors. While LNSS has many advantages, it also has many challenges, one of which is the management of complex renal cystic lesions.

The Bosniak classification system categorizes complex renal cystic lesions into type I, type II, type IIF, type III, and type IV based on CT findings (1). The surgical treatment of complex renal cystic lesions carries inherent risks, including potential malignancy and limitations in the intraoperative frozen pathological examination accuracy, leading to false-negative and false-positive results. In cases where the postoperative pathology reveals malignancy following simple laparoscopic unroofing of a renal cyst, there is a high risk of intraoperative cystic fluid dissemination and tumor implantation. Subsequent radical nephrectomy is often recommended, and the adhesions from the initial operation increase the difficulty and risks of the second operation. For appropriate cases, LNSS can resect the possible malignant renal cystic lesions completely, no matter whether it is benign or malignant, and can preserve the kidney while avoiding associated medical risks (2).

## Methods

### Clinical data

A total of 83 cases of complex renal cysts underwent LNSS in our hospital. Among them, there were 32 men and 51 women, ranging in age from 24 to 73 years (average, 47.22 ± 9.03 years). The diameter of the complex renal cyst ranged from 1.5 to 5.9 cm (average, 3.44 ± 0.86 cm). According to the Bosniak classification, there were 15 cases of type II, 23 cases of type IIF, 29 cases of type III, and 16 cases of type IV complex renal cystic lesions. Based on clinical classification considering the difficulty of the LNSS and the depth of the lesion, the 83 complex renal cystic lesions were categorized into the extra-renal protrusion type (48 cases), the central renal type (15 cases), the renal sinus type (7 cases), and the renal hilum type (13 cases). The RENAL score ranged from 4 to 11, with an average of 7.67 ± 1.73, while the PADUA score ranged from 6 to 13, with an average of 9.48 ± 1.89.

### Clinical classification

**Figure 1:**
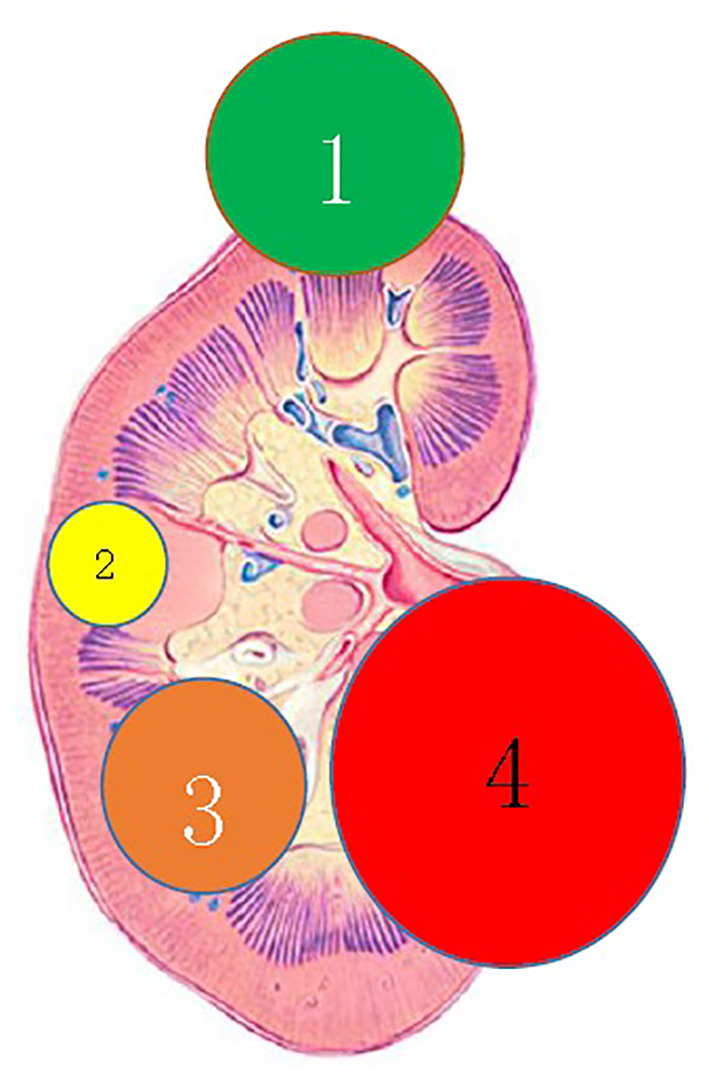
Clinical classification of complex renal cystic lesions. 1) The renal extraocular type; 2) the centrally located type; 3) the renal sinus type; and 4) the renal hilum type.

**Figure 2:**
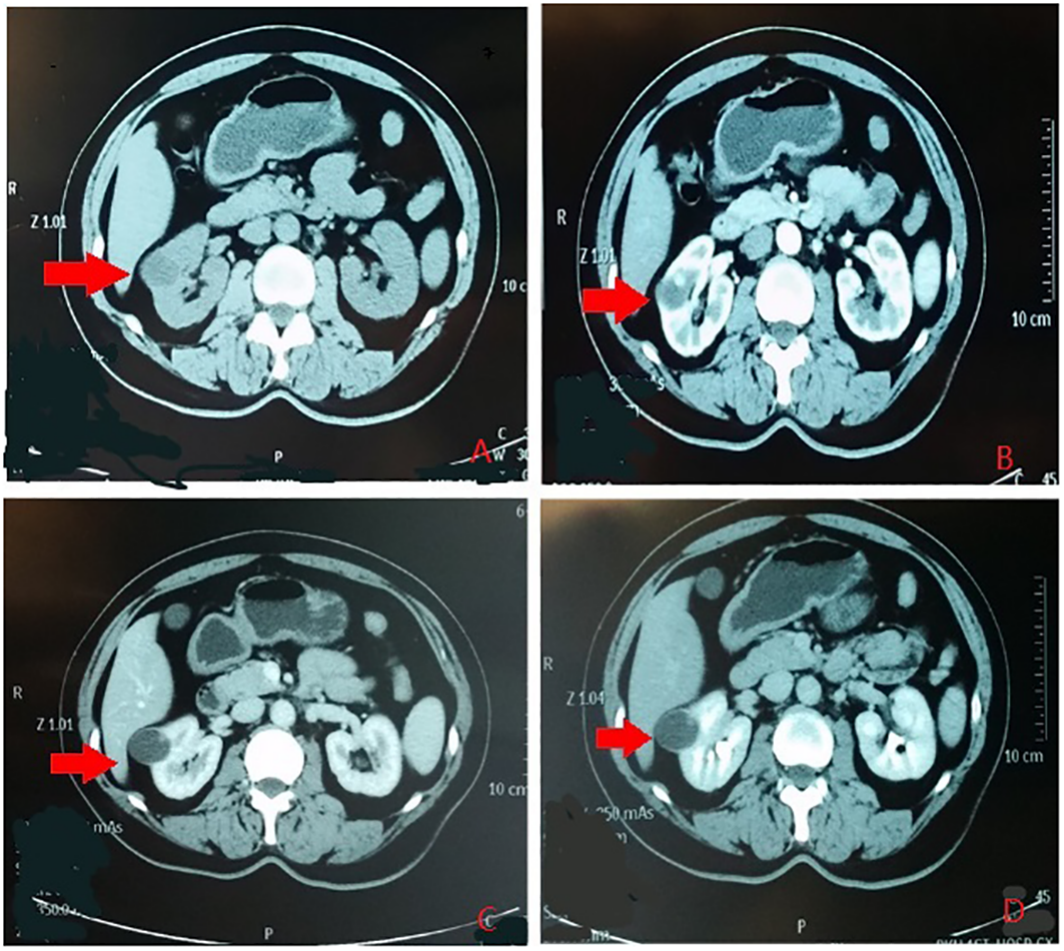
The renal extraocular type. **(A)** Plain scan. **(B)** Arterial phase. **(C)** Venous phase. **(D)** Delayed phase. The red arrows indicates the complex renal cyst.

**Figure 3:**
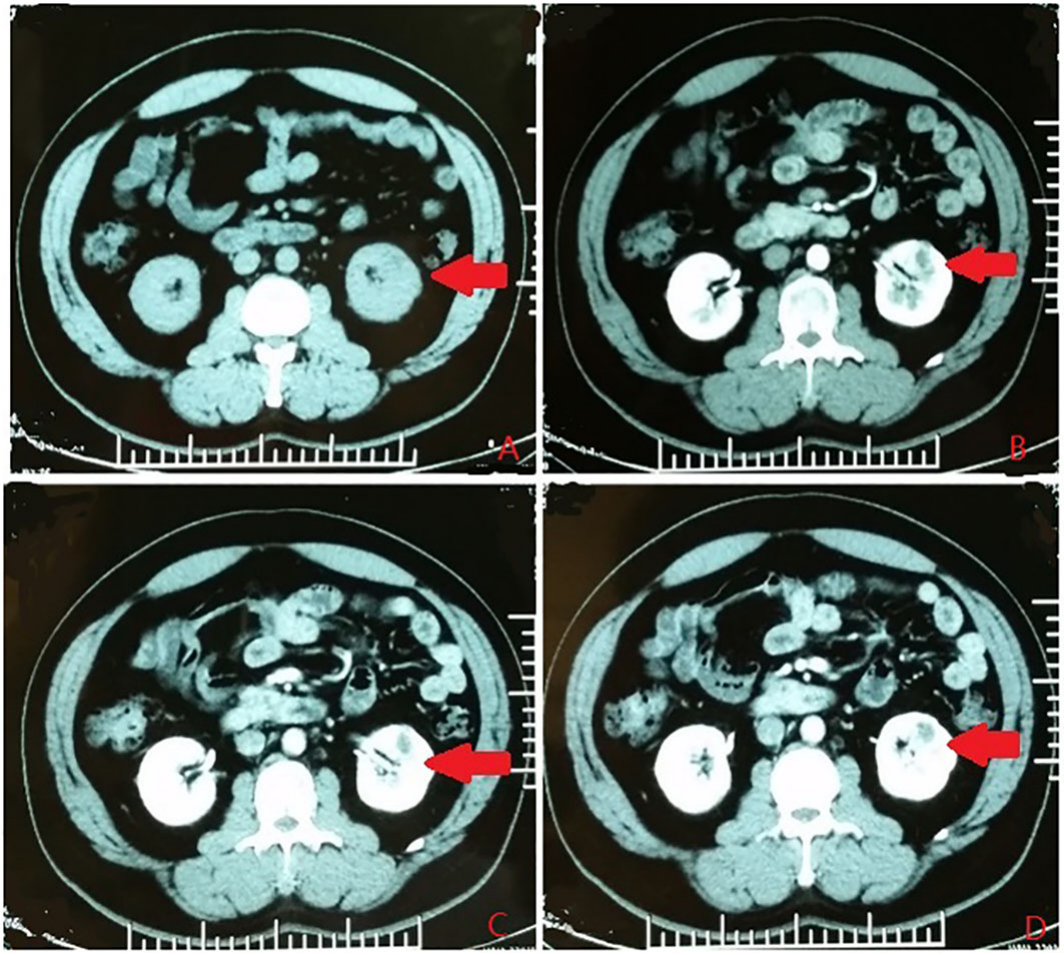
The centrally located type. **(A)** Plain scan. **(B)** Arterial phase. **(C)** Venous phase. **(D)** Delayed phase. The red arrows indicates the complex renal cyst.

**Figure 4:**
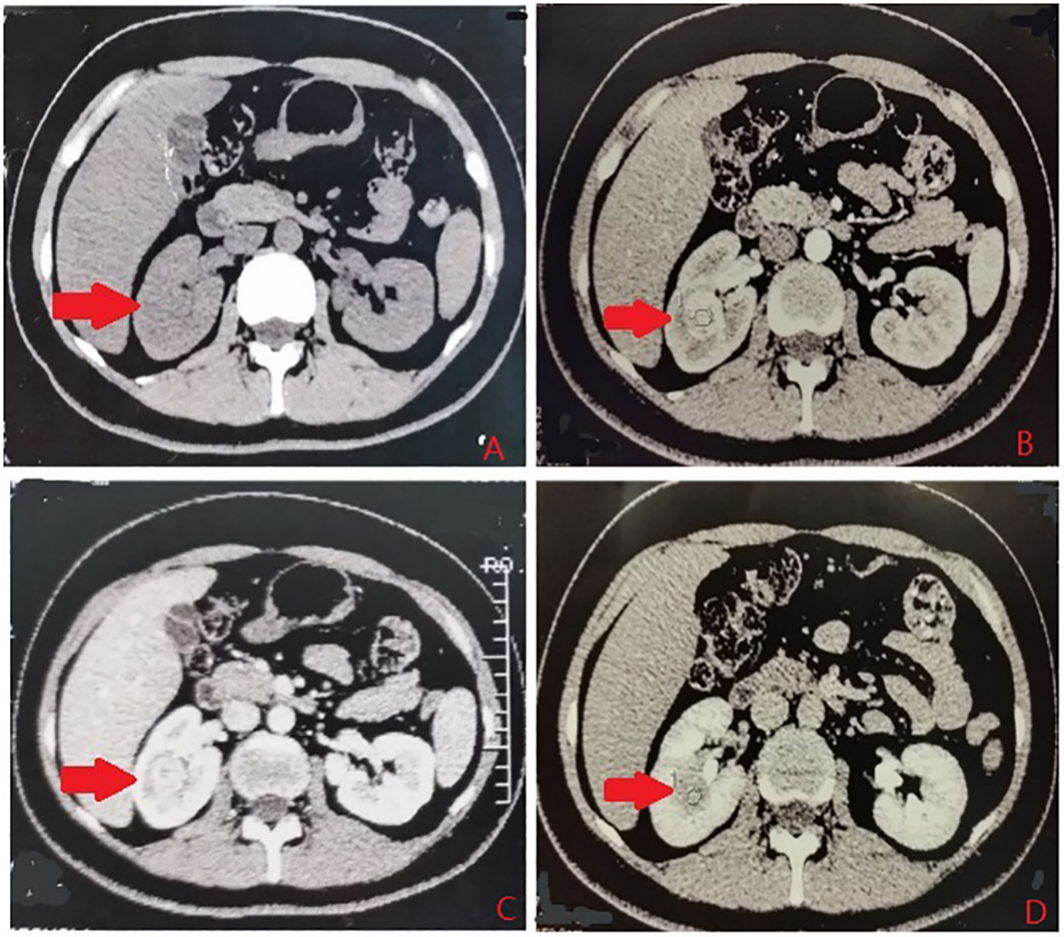
The renal sinus type. **(A)** Plain scan. **(B)** Arterial phase. **(C)** Venous phase. **(D)** Delayed phase. The red arrows indicates the complex renal cyst.

**Figure 5:**
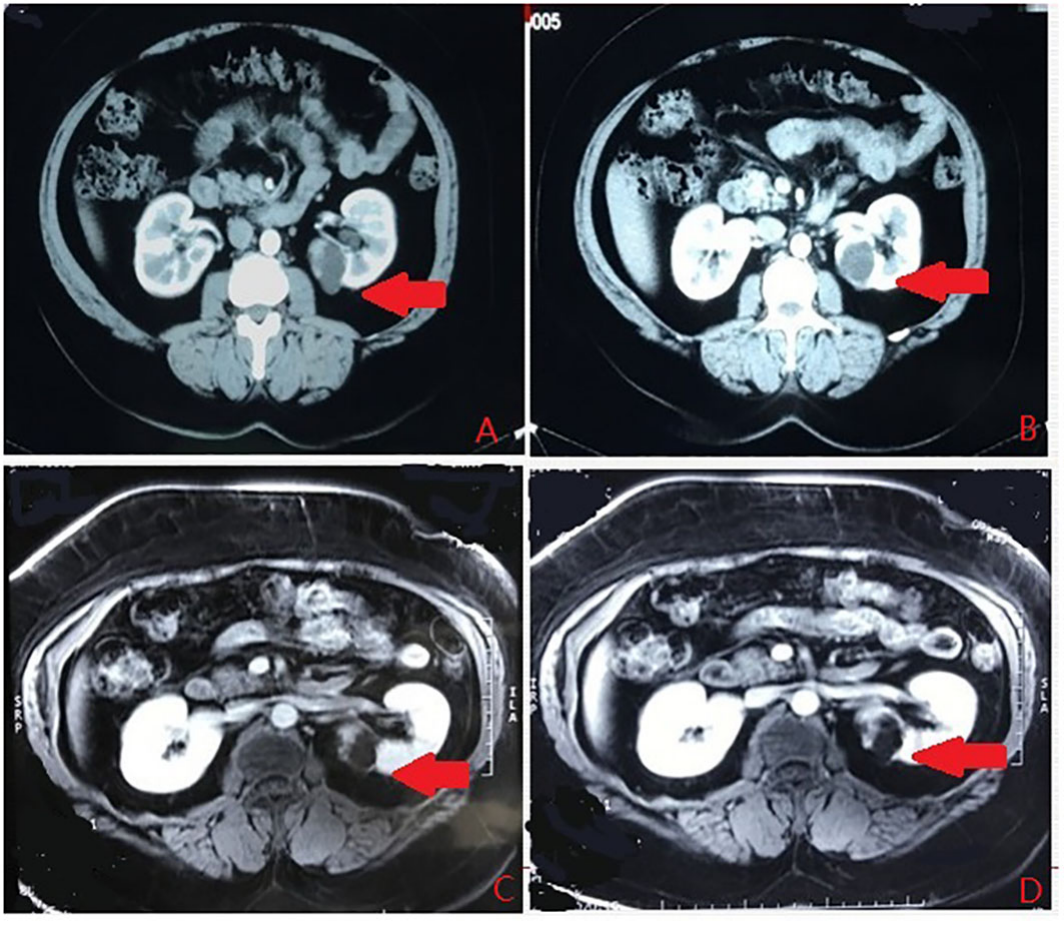
The renal hilum type. **(A)** Arterial phase. **(B)** Vein phase. **(C)** MR image. **(D)** MR image. The arrows indicates the complex renal cyst.

According to the location and complexity of the complex cystic lesions in the kidney, the complex renal cystic lesions can be classified into four types: extra-renal, centrally located, renal sinus, and renal hilum (**Figure 1**). This classification is based on the distance between the renal cyst and the outer surface of the renal parenchyma, as well as the distance between the renal cyst and the inner surface of the renal collecting system. The extra-renal type protrudes from the outer surface of the renal parenchyma without reaching the inner surface of the renal collecting system (**Figure 2**). The centrally located type is situated in the renal parenchyma without obvious outward protrusion, but with a certain distance from the inner surface of the renal collecting system (**Figure 3**). The renal sinus type is located within the renal parenchyma without any obvious outward protrusion, extending into the renal sinus and is very close to the inner surface of the renal collecting system (**Figure 4**). Lastly, the renal hilum type is located on the anterior and posterior lips of the kidney, adjacent to the renal artery, vein, pelvis, and ureter (**Figure 5**).

### Laparoscopic ultrasonography

The Medical TYPE BK 2202 color ultrasonic apparatus was utilized for the renal centrally located type and the renal sinus type of complex renal cystic lesions. The ultrasonic probe is a special probe with a frequency conversion linear array, with frequency of 4–10.0 MHz and an outside diameter of 10.0 mm. It can be adjusted by the handle to achieve axial flexion movement from +90° to −90°. During operation, the ultrasonic probe was coated with a coupling agent and then placed into a sterile sleeve for the laparoscopic procedure (3).

### Surgical procedure

All of the enrolled cases were selected via a retroperitoneal approach in order to ensure that all patients underwent the same type of surgery performed by the same surgeon, reducing selection bias and ensuring the accuracy and comparability of the data. After general anesthesia, the patient was placed in the lateral decubitus position with a waist pad. Three trocars of 12, 10, and 5.5 mm were inserted at the intersection of 2 cm above the iliac crest and the axillary midline, 2 cm below the subcostal margin and the posterior axillary line, and 2 cm below the subcostal margin and the anterior axillary line, respectively. Moreover, a 5.5-mm trocar was added in one case. The retroperitoneal pneumoperitoneum was established and maintained at a pressure of 14 cm H_2_O. Using an ultrasonic knife, a clamp, and a suction device, the kidney was dissected with identification of the renal artery within the peripheral renal fat capsule. The approximate location of the renal tumor was identified based on preoperative imaging. The laparoscopic ultrasonography probe was introduced through the 12-mm trocar to determine the precise locations of the renal centrally located type and renal sinus type lesions, displaying clear visualization of the tumor size and boundary on screen. Following precise delineation of the tumor location and boundary using an electric hook cautery, an arterial occlusion clamp allowed complete resection of the tumor along the outlined margins including the surrounding renal tissue. The inner surface of the renal collecting system and the renal parenchyma were sutured in two layers using an absorbable suture before the release of the arterial occlusion clamp to ensure satisfactory hemostasis (3).

### Data statistics

Data including the duration of operation, bleeding volume, changes in the hemoglobin and serum creatinine levels, and hospitalization time were recorded and analyzed statistically.

## Results

The LNSS was successfully performed on 83 cases of complex renal cystic lesions. The operation time ranged from 35 to 102 min (average, 52.13 ± 14.38 min), the intraoperative bleeding volume ranged from 10 to 200 ml (average, 27.25 ± 12.26 ml), and the renal artery occlusion time ranged from 12 to 28 min (average, 12.46 ± 4.45 min). There were no significant changes in creatinine before and after surgery. The postoperative hospital stay ranged from 5 to 7 days, with an average of 5.8 days.

The postoperative pathological results revealed that there were 71 cases of renal clear cell carcinoma, five cases of low malignant potential multilocular cystic renal tumors, and seven cases of pure renal cysts with all margins negative. For the seven cases of pure renal cysts, six cases were Bosniak type II and one case was Bosniak type IIF. There were no recurrences or metastases after a follow-up of 12–48 months.

## Discussion

The Bosniak classification for complex renal cysts is based on CT findings. Surgical treatment of complex renal cysts carries several risks:

1) Complex renal cysts have the potential for malignancy, while the intraoperative rapid frozen pathology has limited accuracy, leading to the risk of false negatives and false positives.
2) Performing a decapitation surgery on a simple renal cyst carries the risk of tumor dissemination and implantation if the postoperative pathological results reveal malignancy.
3) In cases where a decapitation surgery is performed on a simple renal cyst and malignant pathology is discovered, severe adhesion during subsequent kidney removal surgery could result in high surgical difficulty and significant medical risks.

For appropriate cases of complex renal cysts, LNSS is recommended as it can effectively remove complex renal cysts with potential malignancy while preserving kidney function, regardless of benign or malignant pathological results. Therefore, LNSS can reduce the medical risks associated with complex renal cysts.

The key to a successful LNSS for complex renal cysts lies in the precise location and complete removal of the lesion, without rupture and with negative margins. Surgical management of complex renal cysts within the renal parenchyma presents significant challenges, as excessive removal of normal renal tissue could result in the loss of renal units and potential damage to the collection system, while inadequate tissue resection could lead to residual tumors and positive margins. In cases of complex renal cystic lesions of the renal centrally located type and the renal sinus type, laparoscopic ultrasonography has demonstrated superior performance compared with traditional ultrasound, particularly in assessing the blood supply and the tumor boundary during surgery. The use of intraoperative ultrasound in laparoscopic surgery enables accurate localization and clear resection boundaries for complex renal cysts, ensuring safety and feasibility (4, 5).

The key points of LNSS for complex renal cystic lesions are summarized as follows for the different positions of complex renal cysts:

1) For complex renal cystic lesions of the renal extraocular type, it is necessary to preserve adipose tissue on the surface of the complex renal cyst in order to facilitate intraoperative manipulation. The resection boundary distance should be 0.5 cm from the renal cyst during incision to prevent rupture. The excision depth should be sufficient to ensure that the renal cyst in the renal parenchyma is not compromised. The incision may need to be enlarged appropriately to avoid rupture during the removal process.
2) For complex renal cystic lesions of the renal centrally located type, laparoscopic ultrasonography is necessary for accurate positioning during surgery. It is important to maintain the integrity and tight closure of any collection system breakage if encountered.
3) For complex renal cystic lesions of the renal sinus type, laparoscopic ultrasonography is needed for accurate positioning during laparoscopic surgery. Most often, perforation of the renal collecting system will be required to ensure negative margins. Suturing both the collecting system and the renal parenchyma tightly in two layers is necessary to prevent complications such as urine leakage and bleeding.
4) For the renal hilum type, protection of the renal artery, vein, and ureter at the renal hilum is crucial, and much more attention should be paid during the resection and suturing to reduce potential bleeding and urinary leakage.

Preliminary clinical classification is based on the complexity of the surgical intervention and the depth of the lesion, including the extra-renal type, the centrally located type, the renal sinus type, and the renal hilum type. For the renal extraocular type, complete removal of the complex cystic lesion without rupture is essential. For the centrally located type and the renal sinus type, laparoscopic ultrasonography is necessary for accurate positioning. For the renal sinus type, careful consideration should be given to the potential disruption of the collecting system to ensure negative margins, and tight suturing is necessary to minimize urine leakage and bleeding risk. For the renal hilum type, protection of the renal artery, vein, and ureter at the renal hilum is crucial.

The limitations of this study include the small sample size and the potential selection bias due to its retrospective nature. In sum, LNSS is safe and effective for the removal of complex renal cysts. In appropriate cases, LNSS can completely resect potentially malignant renal cystic lesions, whether benign or malignant, while preserving renal function and minimizing medical risks.

## Data availability statement

The original contributions presented in the study are included in the article/supplementary material. Further inquiries can be directed to the corresponding author.

## Ethics statement

The studies involving humans were approved by PUMCH institutional review board. The studies were conducted in accordance with the local legislation and institutional requirements. Written informed consent for participation was not required from the participants or the participants’ legal guardians/next of kin in accordance with the national legislation and institutional requirements. Written informed consent was obtained from the individual(s) for the publication of any potentially identifiable images or data included in this article.

## Author contributions

DD: Conceptualization, Data curation, Formal analysis, Funding acquisition, Investigation, Methodology, Project administration, Resources, Software, Supervision, Validation, Visualization, Writing – original draft, Writing – review & editing. YZ: Conceptualization, Data curation, Formal analysis, Funding acquisition, Investigation, Methodology, Project administration, Resources, Software, Supervision, Validation, Visualization, Writing – original draft.
